# Blood urea nitrogen to serum albumin ratio predicts 28-day and 90-day mortality in patients with acute pancreatitis: A retrospective cohort study

**DOI:** 10.1371/journal.pone.0335808

**Published:** 2025-10-31

**Authors:** Xu Cai, Xiaoqing Jiang, Wenbin Nan, Zhenyu Peng, Chenlu Wu, Kui Tang

**Affiliations:** 1 Department of Emergency Medical, General Hospital of Ningxia Medical University, Yinchuan, Ningxia, China; 2 Department of Geriatric Respiratory and Critical Care Medicine, Xiangya Hospital, Central South University, Changsha, China; 3 Department of Emergency Medicine, Guilin Hospital of the Second Xiangya Hospital, Central South University, Guilin, China; 4 Department of Emergency Medicine, Second Xiangya Hospital, Central South University, Changsha, China; 5 Department of Cardiology, Second Xiangya Hospital, Central South University, Changsha, China; 6 Department of Ultrasound Diagnosis, The Second Xiangya Hospital, Central South University, Changsha, Hunan, China; Sapporo Medical University, JAPAN

## Abstract

**Background:**

Acute pancreatitis is a prevalent and severe digestive disease with significant mortality. Early identification of high-risk patients is essential for improving outcomes. The ratio of blood urea nitrogen to albumin (BAR) has emerged as a potential prognostic predictor in various critical illnesses. This study explores the associations between BAR and 28-day and 90-day mortality in acute pancreatitis patients.

**Methods:**

This retrospective cohort study enrolled patients diagnosed with acute pancreatitis from the Medical Information Mart for Intensive Care IV (MIMIC-IV) database. BAR was calculated using initial blood urea nitrogen and serum albumin. Patients were categorized into three groups according to the tertiles (T1-T3) of BAR values. Kaplan-Meier survival analysis and restricted cubic spline (RCS) curve assessed the impact of BAR on overall survival. Multivariate Cox regression analysis was used to determine association of BAR with 28-day and 90-day mortality in acute pancreatitis. The receiver operating characteristic (ROC) curve was employed to assess the predictive value of BAR for 28-day and 90-day mortality in acute pancreatitis.

**Results:**

In this study, 452 patients with acute pancreatitis were analyzed, with 28-day mortality rate of 11.7% and 90-day mortality rate of 13.7%. Kaplan-Meier survival analysis indicated a notable increase in mortality rates at 28 days and 90 days among patients with elevated BAR levels (Log-rank *P* < 0.001). Moreover, RCS curve demonstrated a linear and positive relationship between BAR levels and mortality rates at 28 days and 90 days after adjusting all covariates. Multivariate Cox regression analysis identified BAR as an independent risk factor for 28-day and 90-day mortality. Subgroup analysis confirmed a consistent correlation between elevated BAR levels and poor outcomes in acute pancreatitis patients. The area under the curve (AUC) of BAR for predicting 28-day and 90-day mortality was 0.802 and 0.798 respectively, both performing comparably to the Simplified Acute Physiology Score II (SAPS II).

**Conclusion:**

Higher BAR values were significantly associated with increased 28-day and 90-day mortality in acute pancreatitis patients. Moreover, BAR may serve as a simple and effective tool for identifying higher death risk of patients with acute pancreatitis.

## 1. Introduction

Acute pancreatitis is a common acute digestive disease requiring inpatient hospitalization. It is characterized by elevated pancreatic enzymes and the destruction of secretory cells, contributing to systemic inflammatory response syndrome (SIRS) and multiple organ dysfunction syndrome (MODS) [1,2]. Approximately 25% of acute pancreatitis cases develop into severe acute pancreatitis, with a mortality rate reaching to 30% [3,4]. Given its significant impact on public health, identifying high-risk patients with acute pancreatitis early is crucial for adopting appropriate therapeutic approaches and improving outcomes. However, existing scoring systems for acute pancreatitis are often complex and challenging to apply in clinical settings [5–7]. Therefore, there is a pressing need to explore a convenient and validated tool for assessing the prognosis of acute pancreatitis.

Blood urea nitrogen (BUN) is a metabolic byproduct reflecting protein catabolism, nutritional status, and renal function. It has prognostic value in conditions like pneumonia, chronic kidney disease, gastrointestinal hemorrhage, sepsis, and acute pancreatitis [8–11]. Albumin is a major plasma protein with various physiological effects. Several studies have consistently shown that lower albumin is linked to the severity and high mortality of acute pancreatitis [12,13]. Recently, the serum BUN to serum albumin ratio (BAR) has emerged as a useful prognostic indicator of acute kidney injury, acute pulmonary embolism, acute myocardial infarction, and sepsis [14–17]. However, it remains unclear whether BAR predicts 28-day and 90-day mortality of patients with acute pancreatitis. Therefore, in this study, we aimed to investigate the association between BAR and mortality rates at 28 days and 90 days in acute pancreatitis using data from the Medical Information Mart for Intensive Care IV (MIMIC-IV) database.

## 2. Materials and methods

### 2.1 Study design

All data for this retrospective study were sourced from the MIMIC-IV database, an openly accessible critical-care database encompassing over 60,000 intensive care unit (ICU) admissions at Beth Israel Deaconess Medical Center (BIDMC) in Boston, Massachusetts between 2008 and 2019. The use of the MIMIC-IV database was approved by the Institutional Review Boards of the Massachusetts Institute of Technology (Cambridge, MA) and Beth Israel Deaconess Medical Center (Boston, MA). Consent for the original data collection was obtained, and all data used in this study were fully de-identified to protect patient privacy. Informed consent was waived due to de-identification of all data. This study adhered to the Declaration of Helsinki and followed the Strengthening the Reporting of Observational Studies in Epidemiology (STROBE) guidelines.

### 2.2 Study population

**Fig 1:**
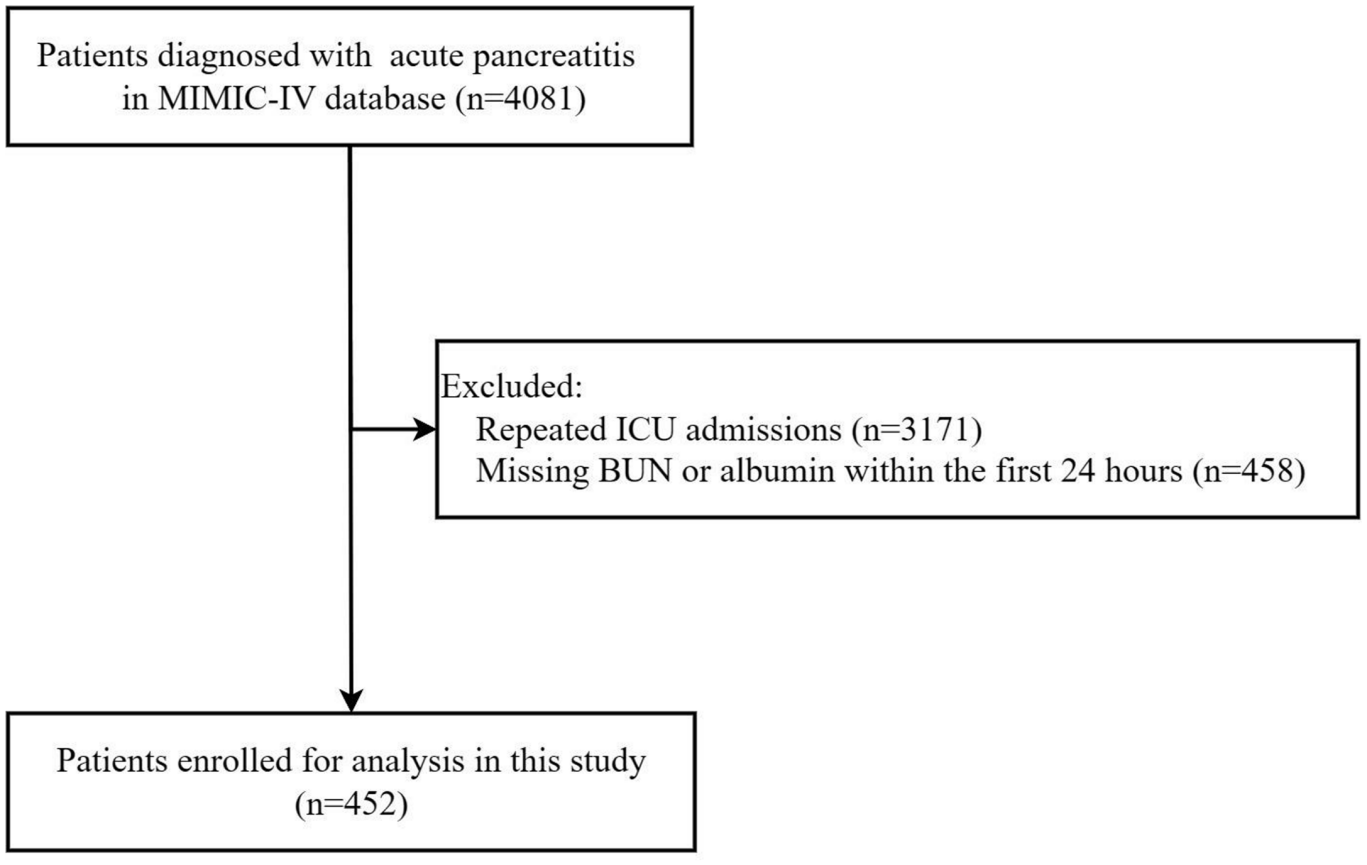
Flow chart of participants’ enrollment in this study. MIMIC-IV, Multiparameter Intelligent Monitoring in Intensive Care IV; ICU, intensive care unit; BUN, blood urea nitrogen.

We enrolled adult patients diagnosed with acute pancreatitis based on International Classification of Diseases, 9th Revision (ICD-9) code 577.0 and 10th Revision (ICD-10) codes K85.00-K85.92 (n = 4081) [12]. The exclusion criteria for the study included: (1) more than one admission in ICU (n = 3171); (2) absence of BUN or albumin data within the first 24 hours of admission (n = 458). Finally, a total of 452 eligible individuals with acute pancreatitis were incorporated into this study as shown in Fig 1. Patients or the public WERE NOT involved in the design, or conduct, or reporting, or dissemination plans of our research. In addition, patients with multiple ICU admissions were excluded to ensure that each individual had only one ICU stay to the dataset, thereby avoiding duplicate records and potential bias from repeated measurements in the same patient.

### 2.3 Data extraction

Data extraction was performed using PostgreSQL tools (v.14) with Navicate Premium software (version 15) from MIMIC-IV database. Extracted variables included: (1) demographic information: age, gender, race, and weight; (2) vital signs: mean arterial pressure (MAP), respiration rate, and heart rate; (3) laboratory indicators: white blood cells (WBC), hemoglobin, platelets, alanine aminotransferase, aspartate aminotransferase, creatinine, serum BUN, albumin, and BAR; (4) intervention measures: vasopressors, renal replacement therapy (RRT), and mechanical ventilation; (5) preexisting conditions: cerebrovascular disease, chronic pulmonary disease, congestive heart failure, myocardial infarction, renal disease, liver disease, diabetes, tumor, and Charlson comorbidity index; (6) critical illness scores: Simplified Acute Physiology Score II (SAPS II); (7) prognosis: length of stay (LOS) in intensive care unit (ICU), LOS in hospital, ICU mortality, in-hospital mortality, 28-day and 90-day mortality. LOS in ICU and hospital was defined as the total duration from ICU or hospital admission to discharge. ICU and in-hospital mortality were determined by survival status at the end of ICU or hospital stay, respectively. The 28-day and 90-day mortality were based on survival status within 28 and 90 days from ICU admission, regardless of discharge status. The BAR (mg/g) was derived by dividing BUN (mg/dL) by serum albumin concentration (g/dL). All laboratory measurements were recorded within the first 24 hours following ICU admission.

### 2.4 Statistical analysis

Continuous variables were presented as mean (standard deviation, SD) for normally distributed variables, or median (interquartile range, IQR) for non-normally distributed variables. Categorical variables are presented as counts and percentages. Baseline characteristics were compared using Student’s t-test, Kruskal-Wallis H test, Chi-squared test, or one-way ANOVA among different groups, as appropriate. Patients with acute pancreatitis were classified into three groups according to BAR tertiles, including T1 group (BAR < 4.7, n = 151), T2 group (4.7 ≤ BAR < 9.68, n = 151) and T3 group (BAR ≥ 9.68, n = 150). Kaplan-Meier survival analysis was plotted to evaluate the impact of BAR on overall survival. The restricted cubic spline (RCS) curve was employed to explore the dose-response relationships between BAR and mortality rates at 28 days and 90 days. Multivariate Cox regression analysis was carried out to determine correlation of BAR with mortality. Three models were constructed to minimize the effects of confounding factors, with adjustments for different covariates. The crude model was adjusted for none covariates. Model I was adjusted for age, gender, race and weight. Model II was adjusted for age, gender, race, weight, mean arterial pressure, respiratory rate, heart rate, WBC, hemoglobin, platelets, alanine aminotransferase, aspartate aminotransferase, creatinine, vasopressor use, renal replacement therapy (RRT), mechanical ventilation, comorbidities, Charlson comorbidity index, and SAPS II score. The discriminative ability of BAR for predicting 28-day and 90-day mortality was evaluated using the area under the receiver operating characteristic curve (AUC-ROC). Additionally, subgroup analyses were performed based on age, gender, vasopressor, renal replacement therapy, and mechanical ventilation. Statistical analyses were performed with Stata (version 15.0) and R software (version 4.2.0). Two-tailed *P* < 0.05 was considered statistically significant.

## 3. Results

### 3.1 Baseline characteristics

The characteristics of patients based on 28-day and 90-day mortality rates were detailed in S1 and S2 Tables, respectively. Of note, the 28-day mortality was 11.7% with 53 non-survivors and 399 survivors, and the 90-day mortality was 13.7% with 62 non-survivors and 390 survivors. At 28 days and 90 days, higher serum BUN and lower albumin levels were found in non-survivors compared to survivors (both, *P* < 0.05). All patients were categorized into three groups based on the tertiles of BAR levels. Among them, statistical differences were observed in age, race, weight, MAP, respiration rate, WBC, hemoglobin, alanine aminotransferase, creatinine, serum BUN, albumin, BAR, vasopressors, renal replacement therapy, mechanical ventilation, congestive heart failure, myocardial infarction, renal disease, Charlson comorbidity index, SAPS II, LOS in ICU, LOS in hospital, ICU mortality, in-hospital mortality, 28-day and 90-day mortality (*P* < 0.05) as demonstrated in Table 1.

**Table 1:** Baseline characteristics of the patients according to BAR values.

	T1 group (n = 151)	T2 group (n = 151)	T3 group (n = 150)	*P-*value
Age (years)	49.43 (39.80, 60.04)	63.03 (50.44, 74.38)	65.29 (52.02, 76.78)	< 0.001
Gender, n (%)				0.071
Female	65 (43.05)	73 (48.34)	53 (35.33)	
Male	86 (56.95)	78 (51.66)	97 (64.67)	
Race, n (%)				0.020
White	99 (65.56)	98 (64.90)	88 (58.67)	
Black/African American	15 (9.93)	14 (9.27)	5 (3.33)	
Others	37 (24.50)	39 (25.83)	57 (38.00)	
Weight (kg)	81.60 (68.15, 99.50)	79.50 (69.00, 97.60)	85.10 (74.02, 105.00)	0.043
MAP (mm Hg)	90.94 (81.51, 101.60)	84.96 (76.68, 96.29)	78.96 (73.22, 88.47)	< 0.001
Respiration rate (bpm)	20.15 (17.07, 23.33)	20.80 (18.59, 24.76)	21.74 (18.45, 24.58)	0.037
Heart rate (bpm)	97.82 ± 18.85	97.26 ± 18.05	95.95 ± 17.45	0.656
WBC (10^9^/L)	11.60 (7.60, 15.75)	13.90 (9.50, 19.25)	13.80 (9.70, 19.77)	0.001
Hemoglobin (g/dL)	11.60 (10.40, 12.80)	11.20 (9.90, 12.60)	10.90 (9.50, 13.00)	0.038
Platelets (10^9^/L)	209.00 (143.50, 281.00)	194.00 (126.50, 271.50)	178.00 (126.00, 240.25)	0.194
Alanine aminotransferase (IU/L)	44.00 (24.00, 134.50)	78.00 (30.00, 203.00)	48.00 (25.00, 116.75)	0.022
Aspartate aminotransferase (IU/L)	61.00 (33.50, 148.00)	87.00 (43.00, 224.50)	75.50 (38.50, 186.25)	0.061
Creatinine (mg/dL)	0.70 (0.60, 0.90)	1.10 (0.80, 1.40)	2.35 (1.60, 4.07)	< 0.001
BUN (mg/dL)	9.00 (7.00, 12.00)	19.00 (16.00, 23.00)	47.00 (37.00, 61.00)	< 0.001
Albumin (g/dL)	3.20 (2.80, 3.60)	3.00 (2.60, 3.30)	2.70 (2.20, 3.20)	< 0.001
BAR (mg/g)	2.94 (2.31, 3.80)	6.76 (5.54, 8.16)	16.91 (12.93, 24.26)	< 0.001
Vasopressors, n (%)	22 (14.57)	50 (33.11)	79 (52.67)	< 0.001
Renal replacement therapy, n (%)	3 (1.99)	22 (14.57)	48 (32.00)	< 0.001
Mechanical ventilation, n (%)	43 (28.48)	80 (52.98)	95 (63.33)	< 0.001
Cerebrovascular disease, n (%)	3 (1.99)	10 (6.62)	11 (7.33)	0.080
Chronic pulmonary disease, n (%)	31 (20.53)	29 (19.21)	31 (20.67)	0.941
Congestive heart failure, n (%)	11 (7.28)	25 (16.56)	27 (18.00)	0.014
Myocardial infarction, n (%)	5 (3.31)	20 (13.25)	20 (13.33)	0.004
Renal disease, n (%)	6 (3.97)	14 (9.27)	38 (25.33)	< 0.001
Liver disease, n (%)	45 (29.80)	44 (29.14)	46 (30.67)	0.959
Diabetes, n (%)	39 (25.83)	39 (25.83)	49 (32.67)	0.313
Tumor, n (%)	10 (6.62)	13 (8.61)	16 (10.67)	0.458
Charlson comorbidity index	3.00 (1.00, 5.00)	4.00 (3.00, 6.00)	5.00 (4.00, 7.00)	< 0.001
SAPS II	25.00 (18.00, 31.00)	36.00 (26.00, 46.00)	47.50 (38.00, 60.00)	< 0.001
LOS in ICU (days)	2.73 (1.21, 4.40)	4.06 (1.83, 9.49)	4.57 (1.94, 13.00)	< 0.001
LOS in hospital (days)	8.28 (5.64, 14.20)	13.16 (7.62, 21.44)	15.42 (9.77, 23.91)	< 0.001
ICU mortality, n (%)	1 (0.66)	11 (7.28)	32 (21.33)	< 0.001
In-hospital mortality, n (%)	1 (0.66)	17 (11.26)	44 (29.33)	< 0.001
28-day mortality, n (%)	1 (0.66)	13 (8.61)	39 (26.00)	< 0.001
90-day mortality, n (%)	1 (0.66)	17 (11.26)	44 (29.33)	< 0.001

BAR, serum BUN to albumin ratio; MAP, mean arterial pressure; WBC, white blood cell; BUN, blood urea nitrogen; SAPS II, Simplified Acute Physiology Score II; LOS, length of stay; ICU, intensive care unit.

### 3.2 Association between BAR levels and 28‑day and 90-day mortality

**Fig 2:**
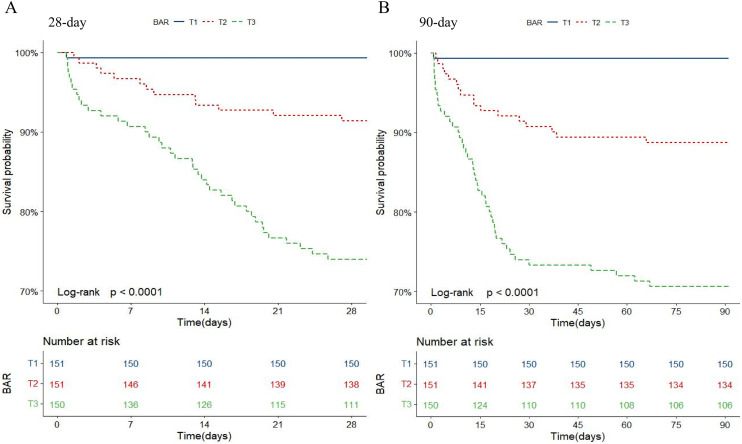
Kaplan–Meier survival curves showing the cumulative 28-day (A) and 90-day (B) mortality in patients with acute pancreatitis stratified by tertiles of BAR. Patients in the highest tertile (T3) exhibited significantly higher mortality compared with T1 and T2 groups (P < 0.001). BAR, blood urea nitrogen to albumin ratio.

**Fig 3:**
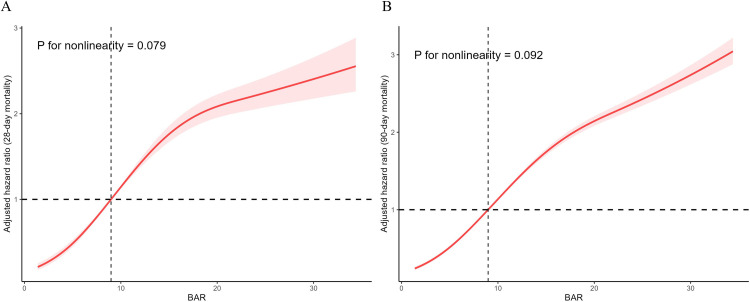
RCS curves illustrating the association between BAR and risk of mortality in patients with acute pancreatitis at 28-day (A) and 90-day (B). The curves were adjusted for all covariates. The figure demonstrated a linear, positive relationship between BAR and mortality risk. RCS, restricted cubic spline; BAR, blood urea nitrogen to albumin ratio.

As shown in Fig 2, Kaplan-Meier survival analysis indicated that acute pancreatitis patients with higher BAR levels had higher mortality rates at 28 days and 90 days among three groups (*P* < 0.001). Moreover, RCS curve demonstrated a linear and positive relationship between BAR levels and mortality rates at 28 days and 90 days in patients with acute pancreatitis after adjusting all covariates (28-day mortality: *P* for non-linearity = 0.079; 90-day mortality: *P* for non-linearity = 0.092) as shown in Fig 3. In addition, to investigate the relationship between BAR levels and mortality rates in acute pancreatitis, three models were constructed using multivariate Cox regression analysis. As depicted in Table 2, BAR levels were positively associated with higher mortality in the Crude model (28-day mortality: HR = 1.08, 95%CI: 1.06–1.11, *P* < 0.001; 90-day mortality: HR = 1.08, 95%CI: 1.06–1.11, *P* < 0.001), Model I (28-day mortality: HR = 1.07, 95%CI:1.05–1.10, *P* < 0.001; 90-day mortality: HR = 1.07, 95%CI: 1.05–1.10, *P* < 0.001), and Model II (28-day mortality: HR = 1.04, 95% CI: 1.01–1.08, *P* = 0.018; 90-day mortality: HR = 1.05, 95% CI:1.02–1.09, *P* = 0.003), respectively. Compared to T1 group, patients in T3 group had increased risks mortality in all three models, with Crude model (28-day mortality: HR = 44.76, 95%CI:6.15–325.84, *P* < 0.001; 90-day mortality: HR = 51.89, 95%CI: 7.15–376.71, *P* < 0.001), Model I (28-day mortality: HR = 32.27, 95%CI: 4.36–239.05, *P* < 0.001; 90-day mortality: HR = 38.20, 95%CI: 5.19–281.44, *P* < 0.001), and Model II (28-day mortality: HR = 12.19, 95%CI: 1.49–99.92, P = 0.020; 90-day mortality: HR = 13.81, 95%CI: 1.74–109.45, P = 0.013), respectively. As shown in S1 Fig, the subgroup analysis indicated that age, gender, vasopressor, renal replacement therapy and mechanical ventilation had no significant interactions with the 28-day and 90-day mortality in acute pancreatitis patients (all, *P* > 0.05).

**Table 2:** Association of BAR with 28-day and 90-day mortality in patients with acute pancreatitis.

	Crude model		Model Ⅰ		Model Ⅱ	
	Crude HR (95%CI)	*P*-value	Adjusted HR (95%CI)	*P*-value	Adjusted HR (95%CI)	*P*-value
28-day mortality						
BAR	1.08 (1.06, 1.11)	< 0.001	1.07 (1.05, 1.10)	< 0.001	1.04 (1.01, 1.08)	0.018
T1 group	Reference		Reference		Reference	
T2 group	13.53 (1.77, 103.41)	0.012	10.27 (1.33, 79.21)	0.026	5.42 (0.66, 44.46)	0.115
T3 group	44.76 (6.15, 325.84)	< 0.001	32.27 (4.36, 239.05)	< 0.001	12.19 (1.49, 99.92)	0.020
*P* for trend	< 0.001		< 0.001		0.003	
90-day mortality						
BAR	1.08 (1.06, 1.10)	< 0.001	1.07 (1.05, 1.10)	< 0.001	1.05 (1.02, 1.09)	0.003
T1 group	Reference		Reference		Reference	
T2 group	17.86 (2.38, 134.20)	0.005	13.41 (1.77, 101.46)	0.012	6.41 (0.81, 50.62)	0.078
T3 group	51.89 (7.15, 376.71)	< 0.001	38.20 (5.19, 281.44)	< 0.001	13.81 (1.74, 109.45)	0.013
*P* for trend	< 0.001		< 0.001		0.001	

The crude model was adjusted for none covariates. Model I was adjusted for age, gender, race and weight. Model II was adjusted for all covariates in this study. BAR, serum BUN to albumin ratio; HR, hazard ratio; CI, confidence interval.

### 3.3 Predictive values of BAR levels for 28-day and 90-day mortality

**Fig 4:**
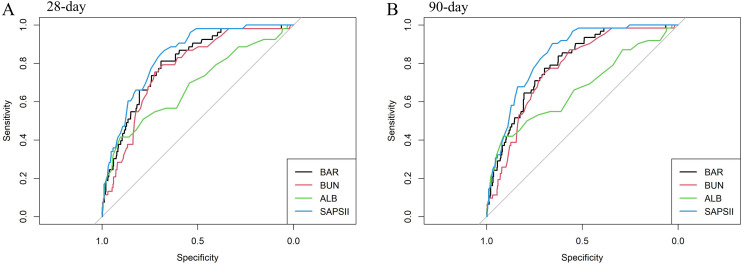
ROC curves for predicting 28-day (A) and 90-day (B) mortality in patients with acute pancreatitis. The predictive performance of BAR was compared with serum BUN, ALB, and SAPS II. The AUC of BAR was similar to that of SAPS II and higher than those of BUN and ALB. ROC, receiver operating characteristic; BAR, blood urea nitrogen to albumin ratio; BUN, blood urea nitrogen; ALB, albumin; SAPS II, Simplified Acute Physiology Score II.

As shown in Fig 4 and Table 3, ROC curve was plotted to assess the predictive values of BAR level, serum BUN, albumin, and SAPS II for 28-day and 90-day mortality in acute pancreatitis. For prediction of mortality at 28-day, area under the curve (AUC) was 0.802 (95% CI:0.743–0.860) for BAR, 0.774 (95% CI:0.713–0.834) for serum BUN, 0.683 (95% CI:0.599–0.768) for albumin, and 0.841 (95% CI:0.792–0.889) for SAPSII, respectively. Meanwhile, for prediction of mortality at 90-day, AUC was 0.798 (95% CI:0.744–0.852) for BAR, 0.773 (95% CI:0.717–0.829) for serum BUN, 0.669 (95% CI:0.588–0.751) for albumin, and 0.846 (95% CI:0.803–0.890) for SAPSII, respectively. Compared to BAR, the predictive values of serum BUN and albumin levels were obviously lower for 28-day mortality (BUN: *P* = 0.010; ALB: *P* = 0.010) and 90-day mortality (BUN: *P* = 0.015; ALB: *P* = 0.002). There was no significant difference between BAR and SAPSII in predicting 28-day mortality (*P* = 0.226) and 90-day mortality ( = 0.105).

**Table 3:** Information of ROC curves for predicting mortality of acute pancreatitis.

Variables	AUC	95%CI	*P*-value
28-day mortality			
BAR	0.802	0.743, 0.860	Reference
BUN	0.774	0.713, 0.834	0.010
ALB	0.683	0.599, 0.768	0.010
SAPSII	0.841	0.792, 0.889	0.226
90-day mortality			
BAR	0.798	0.744, 0.852	Reference
BUN	0.773	0.717, 0.829	0.015
ALB	0.669	0.588, 0.751	0.002
SAPSII	0.846	0.803, 0.890	0.105

BAR, serum BUN to albumin ratio; BUN, blood urea nitrogen; ALB, albumin; SAPS II, Simplified Acute Physiology Score II; AUC, area under the curve; CI, confidence interval.

## 4. Discussion

Acute pancreatitis is a critical condition marked by damage to the acinar cells. Despite many advances in diagnosis and treatment, the mortality rate of acute pancreatitis remains high [18,19]. Thus, early identification of high-risk patients remains crucial. In this study, we found that BAR was significantly higher in non-survivors compared to survivors at 28 days and 90 days. Moreover, Kaplan-Meier survival analysis indicated that 28-day and 90-day mortality of acute pancreatitis in T3 group was paramount. Furthermore, BAR showed a linear positive association with 28-day and 90-day mortality after adjustment for covariates. In addition, BAR had predictive effects on the 28-day and 90-day mortality of acute pancreatitis. Therefore, our findings provide evidence that BAR is an available predictor for the outcome of acute pancreatitis.

Severe acute pancreatitis is often complicated by multiple organ failure and remains associated with high mortality. Early identification of high-risk patients is essential to guide intensive care and optimize outcomes [20]. Up to now, numerous clinical biomarkers, including age, body temperature, WBC, platelets, BUN, SpO2, hemoglobin, platelet-lymphocyte ratio (PLR), neutrophil-lymphocyte ratio (NLR), and red-cell distribution width (RDW), are associated with the severity and mortality of patients with acute pancreatitis [8,19]. However, targeting these risk factors alone has not been sufficient to reduce mortality in acute pancreatitis. Recent studies have reported that BAR is linked to the short-term outcomes and mortality in critically ill patients [21–23]. Wang et al. found that higher BAR value was related with higher mortality of septic patients admitted to ICU, which was regarded as an independent risk factor for death [17]. Our findings demonstrated that BAR values had a linear and positive correlation with 28-day and 90-day mortality in patients with acute pancreatitis. Thus, an elevated BAR value serves as an independent risk factor for the mortality of patients with acute pancreatitis.

Although traditional scoring systems such as Acute Physiology and Chronic Health Evaluation (APACHE) II score, Bedside Index for the Severity in Acute Pancreatitis (BISAP), albumin-bilirubin (ALBI) score, and SAPS II are available to stratify disease severity, their complexity may limit real-time applicability. In this context, BAR offers a simpler, rapidly accessible alternative for early prognostication [5,24–26]. However, these predictive methods are complex, time-consuming and have variable accuracy, which limit their practicality in clinical settings. Therefore, it is important to explore simple and efficient predictive methods for acute pancreatitis. Recently, BAR has emerged as a novel predictor for the mortality of community-acquired pneumonia, acute respiratory failure, Escherichia coli bacteremia and acute kidney injury. However, no studies have yet used BAR to predict the mortality of acute pancreatitis [14,27–29]. In this study, BAR is proved to be a simple predictor of 28- and 90-day mortality in acute pancreatitis.

Emerging evidence suggests that inflammation plays a central role in the progression of acute pancreatitis by driving acinar cell necrosis and systemic complications [30]. Given the immunological relevance of both albumin and BUN, BAR may serve not only as a prognostic marker but also reflect underlying inflammatory burden [31–33]. In acute pancreatitis, the powerful pro-inflammatory immune response amplifies the pancreatic injury and inflammatory cascades, finally resulting in SIRS and MODS [34]. Increasing evidence demonstrates that albumin prompts the production of multiple anti-inflammatory agents including lipoxins, hemolysins, and protective proteins [12]. Shannon et al. revealed that lower albumin was positively associated with higher risk of both hospitalization and death in various diseases, which might be mediated by inflammation [35]. BUN is not only a marker of renal function, but also a predictor of systemic immune-inflammatory status. Guo et al. found that BUN level had a negative correlation with Systemic Immune Inflammatory Index (SII) [36]. Therefore, we speculated that inflammation might be involved in the association between BAR and poor outcomes of acute pancreatitis.

The main advantages of this study were as follows. Firstly, this was the first study to explore the relationship between BAR and 28-day and 90-day mortality in patients with acute pancreatitis, addressing a significant gap in the current research. Secondly, the patients of our study were sourced from MIMIC-IV, which offered a representative sample of American adults with acute pancreatitis. This enhanced the generalizability of our findings. Thirdly, we adjusted for various potential confounding factors, which increased the reliability of our findings. Nevertheless, our study had some limitations. First, a single measurement of BAR might not fully reflect dynamic changes during disease progression. Serial measurements may offer better prognostic value. Second, due to the limited number of events, the hazard ratios for mortality of acute pancreatitis should be interpreted with caution. Third, owing to the inherent limitations of the MIMIC-IV database, the etiologies of acute pancreatitis were largely unavailable. Fourth, the severity of acute pancreatitis could not be evaluated using conventional imaging-based scoring systems, as contrast-enhanced radiologic data were not accessible in MIMIC-IV database. Fifth, the exact cause of death was not documented in the database, preventing differentiation between mortality directly related to pancreatitis and that due to other comorbidities. Lastly, although we adjusted for many known prognostic indicators, several potentially important variables (e.g., imaging findings, nutritional status, inflammatory cytokine levels, and pre-ICU treatments) were not available, which may have introduced residual confounding. Future prospective studies with more comprehensive clinical and biological data are warranted to validate and extend our findings.

## 5. Conclusion

BAR was positively associated with 28-day and 90-day mortality in patients with acute pancreatitis. Moreover, BAR was an accessible and effective prognostic tool for the mortality of acute pancreatitis. However. future research should be performed to validate its predictive power.

## Supporting information

S1 Fig Forest plots for subgroup analyses of the association between BAR and mortality at 28-day (A) and 90-day (B) in patients with acute pancreatitis. Subgroups were stratified by age, sex, vasopressor use, RRT, and mechanical ventilation. The results indicated that elevated BAR consistently predicted higher mortality risk across all subgroups, with no significant interactions detected. BAR, blood urea nitrogen to albumin ratio; RRT, renal replacement therapy.
(TIF)

S1 Table Characteristics of patients based on 28-day mortality. MAP, mean arterial pressure; WBC, white blood cell; BUN, blood urea nitrogen; BAR, serum BUN to albumin ratio; SAPS II, Simplified Acute Physiology Score II.
(DOCX)

S2 Table Characteristics of patients based on 90-day mortality. MAP, mean arterial pressure; WBC, white blood cell; BUN, blood urea nitrogen; BAR, serum BUN to albumin ratio; SAPS II, Simplified Acute Physiology Score II.
(DOCX)
