# Supplementary figures and images for: Blood urea nitrogen to serum albumin ratio predicts 28-day and 90-day mortality in patients with acute pancreatitis: A retrospective cohort study
Source: PLoS One. 2025 Oct 31;20(10):e0335808. doi: 10.1371/journal.pone.0335808 (PMC12578258; doi:10.1371/journal.pone.0335808)

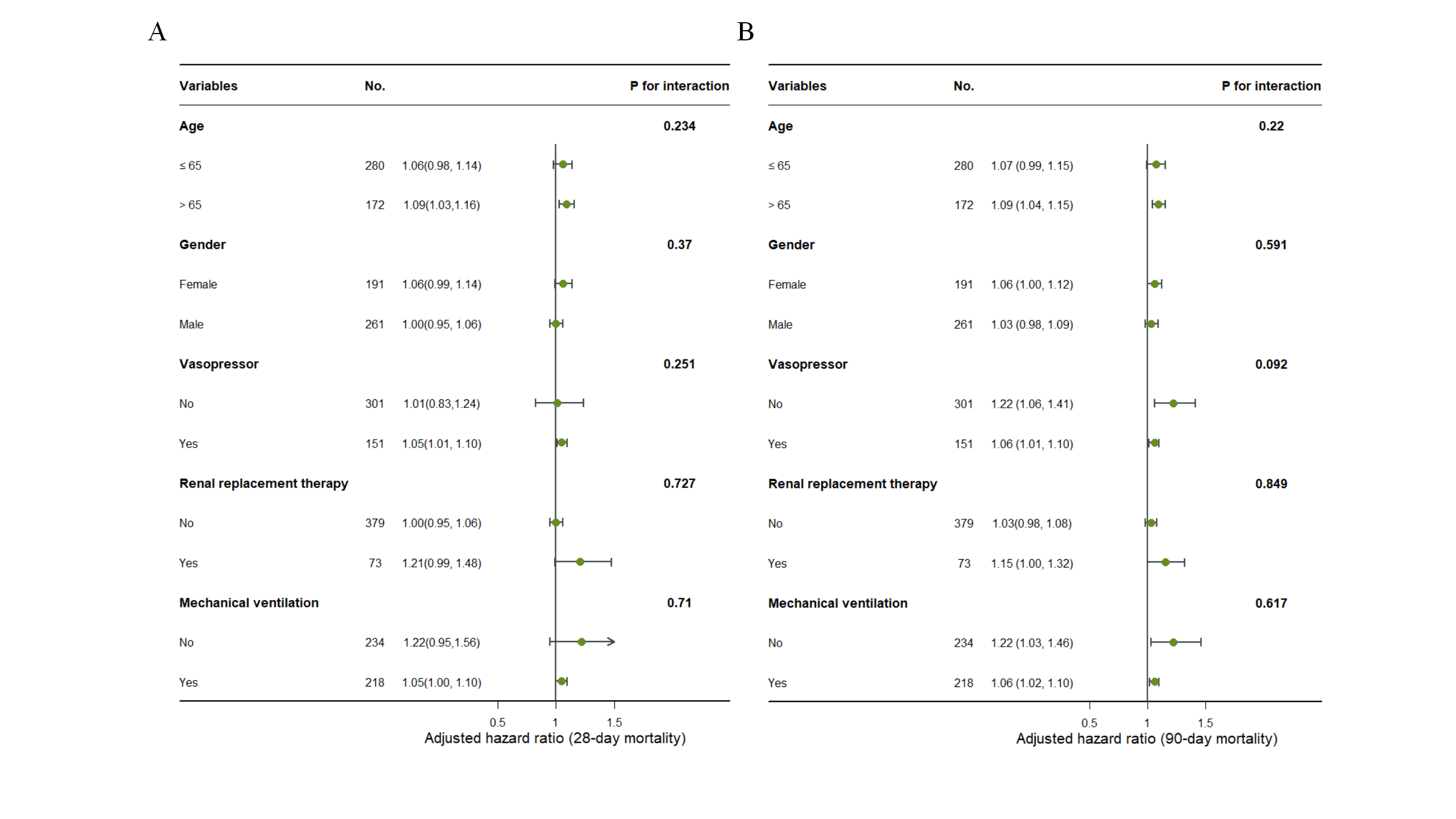

Supplement: S1 Fig — Subgroups were stratified by age, sex, vasopressor use, RRT, and mechanical ventilation. The results indicated that elevated BAR consistently predicted higher mortality risk across all subgroups, with no significant interactions detected. BAR, blood urea nitrogen to albumin ratio; RRT, renal replacement therapy. (TIF) [file pone.0335808.s001.tif]
