# Supplementary material for: Blood urea nitrogen to serum albumin ratio predicts 28-day and 90-day mortality in patients with acute pancreatitis: A retrospective cohort study
Source: PLoS One. 2025 Oct 31;20(10):e0335808. doi: 10.1371/journal.pone.0335808 (PMC12578258; doi:10.1371/journal.pone.0335808)
Supplement: S1 Table — MAP, mean arterial pressure; WBC, white blood cell; BUN, blood urea nitrogen; BAR, serum BUN to albumin ratio; SAPS II, Simplified Acute Physiology Score II. (DOCX) [file pone.0335808.s002.docx]

**Supplementary Table 1** Characteristics of patients based on 28-day mortality

| Variable | Total  (n = 452) | Survivors  (n = 399) | Non-survivors  (n = 53) | *P*-value |
| --- | --- | --- | --- | --- |
| Age (years) | 58.23 (46.32, 72.34) | 56.42 (45.63, 70.93) | 67.77 (58.19, 80.40) | **< 0.001** |
| Gender, n (%) |  |  |  | 0.858 |
| Female | 191 (42.26) | 168 (42.11) | 23 (43.40) |  |
| Male | 261 (57.74) | 231 (57.89) | 30 (56.60) |  |
| Race, n (%) |  |  |  | **0.011** |
| White | 285 (63.05) | 259 (64.91) | 26 (49.06) |  |
| Black/African American | 34 (7.52) | 32 (8.02) | 2 (3.77) |  |
| Others | 133 (29.42) | 108 (27.07) | 25 (47.17) |  |
| Weight (kg) | 82.65 (70.65, 100.35) | 82.30 (70.85, 100.55) | 84.80 (70.50, 96.00) | 0.815 |
| MAP (mmHg) | 84.95 (76.44, 96.19) | 86.73 (77.60, 97.47) | 75.78 (68.98, 82.17) | **< 0.001** |
| Respiration rate (bpm) | 20.62 (18.25, 24.39) | 20.48 (18.00, 24.16) | 22.22 (18.93, 25.14) | **0.044** |
| Heart rate (bpm) | 97.01 ± 18.10 | 97.18 ± 18.03 | 95.76 ± 18.74 | 0.593 |
| WBC (10^9^/L) | 13.10 (8.78, 18.42) | 12.90 (8.80, 18.35) | 14.30 (8.70, 18.50) | 0.566 |
| Hemoglobin (g/dL) | 11.20 (9.90, 12.83) | 11.30 (10.00, 12.90) | 10.50 (9.50, 12.30) | **0.024** |
| Platelets (10^9^/L) | 189.50 (133.00, 270.25) | 194.00 (137.50, 272.00) | 135.00 (88.00, 219.00) | **< 0.001** |
| Alanine aminotransferase (IU/L) | 54.00 (25.00, 168.50) | 53.00 (25.00, 167.50) | 61.00 (32.00, 173.00) | 0.301 |
| Aspartate aminotransferase (IU/L) | 77.00 (36.75, 181.75) | 71.00 (35.00, 166.00) | 138.00 (50.00, 300.00) | **0.003** |
| Creatinine (mg/dL) | 1.05 (0.70, 1.90) | 1.00 (0.70, 1.70) | 1.80 (1.10 , 3.10) | **< 0.001** |
| Serum BUN (mmol/L) | 19.00 (12.00, 37.00) | 18.00 (11.00, 32.00) | 40.00 (29.00, 56.00) | **< 0.001** |
| Albumin (g/L) | 3.00 (2.57, 3.40) | 3.00 (2.60, 3.40) | 2.50 (2.10, 3.10) | **< 0.001** |
| BAR | 6.71 (3.82, 12.90) | 6.00 (3.57, 10.75) | 16.45 (9.63, 25.26) | **< 0.001** |
| Vasopressors, n (%) | 151 (33.41) | 112 (28.07) | 39 (73.58) | **< 0.001** |
| Renal replacement therapy, n (%) | 73 (16.15) | 51 (12.78) | 22 (41.51) | **< 0.001** |
| Mechanical ventilation, n (%) | 218 (48.23) | 178 (44.61) | 40 (75.47) | **< 0.001** |
| Cerebrovascular disease, n (%) | 24 (5.31) | 19 (4.76) | 5 (9.43) | 0.272 |
| Chronic pulmonary disease, n (%) | 91 (20.13) | 83 (20.80) | 8 (15.09) | 0.330 |
| Congestive heart failure, n (%) | 63 (13.94) | 55 (13.78) | 8 (15.09) | 0.796 |
| Myocardial infarction, n (%) | 45 (9.96) | 37 (9.27) | 8 (15.09) | 0.184 |
| Renal disease, n (%) | 58 (12.83) | 50 (12.53) | 8 (15.09) | 0.600 |
| Liver disease, n (%) | 135 (29.87) | 104 (26.07) | 31 (58.49) | **< 0.001** |
| Diabetes, n (%) | 127 (28.1) | 115 (28.82) | 12 (22.64) | 0.347 |
| Tumor, n (%) | 39 (8.63) | 28 (7.02) | 11 (20.75) | 0.002 |
| Charlson comorbidity index | 4.00 (2.00, 6.00) | 4.00 (2.00, 6.00) | 6.00 (5.00, 8.00) | **< 0.001** |
| SAPS II | 35.00 (25.00, 48.00) | 32.00 (24.00, 44.00) | 55.00 (44.00, 71.00) | **< 0.001** |

MAP, mean arterial pressure; WBC, white blood cell; BUN, blood urea nitrogen; BAR, serum BUN to albumin ratio; SAPS II, Simplified Acute Physiology Score II.
