# Supplementary material for: Blood urea nitrogen to serum albumin ratio predicts 28-day and 90-day mortality in patients with acute pancreatitis: A retrospective cohort study
Source: PLoS One. 2025 Oct 31;20(10):e0335808. doi: 10.1371/journal.pone.0335808 (PMC12578258; doi:10.1371/journal.pone.0335808)
Supplement: S2 Table — MAP, mean arterial pressure; WBC, white blood cell; BUN, blood urea nitrogen; BAR, serum BUN to albumin ratio; SAPS II, Simplified Acute Physiology Score II. (DOCX) [file pone.0335808.s003.docx]

**Supplementary Table 2** Characteristics of patients based on 90-day mortality

| Variable | Total  (n = 452) | Survivors  (n = 390) | Non-survivors  (n = 62) | *P*-value |
| --- | --- | --- | --- | --- |
| Age (years) | 58.23 (46.32, 72.34) | 55.74 (45.53, 70.39) | 69.24 (58.69, 80.36) | **< 0.001** |
| Gender, n (%) |  |  |  | 0.524 |
| Female | 191 (42.26) | 162 (41.54) | 29 (46.77) |  |
| Male | 261 (57.74) | 228 (58.46) | 33 (53.23) |  |
| Race, n (%) |  |  |  | **0.038** |
| White | 285 (63.05) | 254 (65.13) | 31 (50) |  |
| Black/African American | 34 (7.52) | 30 (7.69) | 4 (6.45) |  |
| Others | 133 (29.42) | 106 (27.18) | 27 (43.55) |  |
| Weight (kg) | 82.65 (70.65, 100.35) | 82.14 (70.55, 100.45) | 85 (71.12, 96.38) | 0.850 |
| MAP (mmHg) | 84.95 (76.44, 96.19) | 86.81 (78.14, 97.63) | 74.85 (69.03, 81.79) | **< 0.001** |
| Respiration rate (bpm) | 20.62 (18.25, 24.39) | 20.48 (17.86, 24.15) | 22.22 (19.26, 25.18) | **0.016** |
| Heart rate (bpm) | 97.01 ± 18.1 | 97.26 ± 18.13 | 95.43 ± 18 | 0.459 |
| WBC (10^9^/L) | 13.1 (8.78, 18.42) | 12.85 (8.8, 18.18) | 14.9 (8.77, 19.15) | 0.279 |
| Hemoglobin (g/dL) | 11.2 (9.9, 12.83) | 11.3 (10.03, 12.9) | 10.5 (9.05, 12.52) | **0.014** |
| Platelets (10^9^/L) | 189.5 (133, 270.25) | 193 (136.25, 271.75) | 147.5 (94.75, 260) | **0.005** |
| Alanine aminotransferase (IU/L) | 54 (25, 168.5) | 52 (25, 167.75) | 60 (32, 171) | 0.288 |
| Aspartate aminotransferase (IU/L) | 77 (36.75, 181.75) | 70.5 (35, 164) | 132.5 (50.5, 277.25) | **0.002** |
| Creatinine (mg/dL) | 1.05 (0.7, 1.9) | 1 (0.7, 1.7) | 1.75 (1.1, 3) | **< 0.001** |
| Serum BUN (mmol/L) | 19 (12, 37) | 17 (11, 30.75) | 40 (26.25, 55) | **< 0.001** |
| Albumin (g/L) | 2.98 ± 0.63 | 3.04 ± 0.6 | 2.63 ± 0.7 | **< 0.001** |
| BAR | 6.71 (3.82, 12.9) | 5.9 (3.53, 10.49) | 15.42 (9.09, 24.95) | **< 0.001** |
| Vasopressors, n (%) | 151 (33.41) | 106 (27.18) | 45 (72.58) | **< 0.001** |
| Renal replacement therapy, n (%) | 73 (16.15) | 47 (12.05) | 26 (41.94) | **< 0.001** |
| Mechanical ventilation, n (%) | 218 (48.23) | 171 (43.85) | 47 (75.81) | **< 0.001** |
| Cerebrovascular disease, n (%) | 24 (5.31) | 19 (4.87) | 5 (8.06) | 0.354 |
| Chronic pulmonary disease, n (%) | 91 (20.13) | 80 (20.51) | 11 (17.74) | 0.738 |
| Congestive heart failure, n (%) | 63 (13.94) | 53 (13.59) | 10 (16.13) | 0.735 |
| Myocardial infarction, n (%) | 45 (9.96) | 35 (8.97) | 10 (16.13) | 0.129 |
| Renal disease, n (%) | 58 (12.83) | 50 (12.82) | 8 (12.9) | 0.999 |
| Liver disease, n (%) | 135 (29.87) | 103 (26.41) | 32 (51.61) | **< 0.001** |
| Diabetes, n (%) | 127 (28.1) | 114 (29.23) | 13 (20.97) | 0.233 |
| Tumor, n (%) | 39 (8.63) | 28 (7.18) | 11 (17.74) | **0.012** |
| Charlson comorbidity index | 4.00 (2.00 , 6.00) | 4.00 (2.00 , 6.00) | 6.00 (5.00 , 8.00) | **< 0.001** |
| SAPS II | 35 (25, 48) | 32 (23, 43) | 55 (44, 69.5) | **< 0.001** |

MAP, mean arterial pressure; WBC, white blood cell; BUN, blood urea nitrogen; BAR, serum BUN to albumin ratio; SAPS II, Simplified Acute Physiology Score II.
